# Supplementary material for: Relationship between grammar and schizophrenia: a systematic review and meta-analysis
Source: Commun Med (Lond). 2025 Jun 16;5:235. doi: 10.1038/s43856-025-00944-1 (PMC12170843; doi:10.1038/s43856-025-00944-1)
Supplement: Supplementary file 2 — Supplementary Material [file 43856_2025_944_MOESM2_ESM.pdf]

Table of contents

Supplementary Note 1: Full search terms for PUBMED database

Supplementary Note 2: Illness Severity Index

Supplementary Note 3: Domain-specific choices of variables

Supplementary Note 1: Full search terms for PUBMED database

((((schizophreni* OR schizo* OR psychot* OR psychos*) OR (exp schizophrenia/ OR exp "schizophrenia spectrum and other psychotic disorders"/))) AND ((language OR verbal OR linguistic OR speech OR communicat* OR thought) OR (exp language/ OR exp verbal behavior/ OR exp speech/ OR exp communication/ OR exp cognition/))) AND ((syntax OR syntactic OR grammar*) OR (exp syntax/ OR exp grammar/ OR exp "linguistics"/)))

Supplementary Note 2: Illness Severity Index

Various studies that we identified for this review used different scales to quantify the overall burden (positive plus negative symptoms) of schizophrenia. We opted to convert each total score into a common metric varying between 0 to 1, where 1 represents the maximum possible score on the rating scale, and 0 represents the minimum possible value. To do this, we used the mean score reported in each patient cohort and subtracted either 0 (SANS and SAPS, as the minimum possible score is zero) or the number of items for scales with minimum item score as 1 [PANSS-8, PANSS, BPRS, BPRS-E] from the mean scores, divided by maximum minus minimum scores possible per scale (total items multiplied by maximum score per item minus 0 or the total number of items with score of 1 as minimum). We derived these severity scores separately for sub-samples with FTD or no-FTD, first episode, or established schizophrenia whenever such a split was reported, as long as the averages came from the same scale, covering the full patient cohort in each study. This approach was not used when only positive or negative domain scores or FTD scores alone were reported, as neither of them, on their own, capture the full severity of the illness. Thus, our illness severity index provided a comparable metric to study covariance with syntax-related effect sizes across the samples. It is important to note that these values should not be considered absolute measures of illness burden, but merely an index that allows for some comparability across heterogeneous clinical samples.

Supplementary Note 3: Domain-specific choices of variables

Studies reporting syntactic anomaly/error detection often tested other types of errors (semantic, pragmatic as well as non-anomalous sentences). For meta-analytic synthesis, we extracted the error/accuracy rates for sentences with morphosyntactic violations. Studies reporting syntactic comprehension reported comprehension of less complex sentences (e.g., subject-relative or short form sentences[1], short version of the Token test[2]; we extracted the performance metrics for stimuli with sufficient syntactic complexity within each study (e.g., Test for the Reception of Grammar[3], non-canonical sentences[4], object-relative sentences at conversational speed[5], paired rather than single sentences[6]), reported in the full rather than any selected sub-samples [7,8]. If more than one variable measuring the same construct with the same approach were to be found in a single study, averages were taken as summary measures for the analysis (long sentences and object-relative clauses[1]; across tasks[9,10]).

Studies reporting global complexity in production often reported divergent variables; in these cases, we selected the primary measure of interest for each study (e.g., complex[11], simple/matrix sentences[9,12,13]) and those that captured global aspects across the discourse whenever available (e.g., fully formed sentences with clausal structure[14], proportion of sentences with embedded clauses[15]). Derived scores (e.g., principal component factors) were discarded in favor of the individual items forming the construct of interest (e.g., sentences with embedded clauses instead of ‘complexity’ factor scores[16]).

Specific indices for phrasal complexity varied across studies; in most cases, the authors had no specific expectations as to the relative importance of one variable over the other. In these cases, we selected those that were specific to clausal structure (complement clauses rather than the number of grammatical dependents[17,18]) and those that were measured with minimal variability in the control group (e.g., tensed complements without complementizer or conjoined clauses[19,20]).

For production length contrasts, we included both mean length of sentences as well as utterances as long as the measurements were done in the same manner in both patients and control groups. We excluded mean turn length[21] as this measure is influenced by the interviewer’s conversational behaviour. Measures based on unambiguous stimuli (e.g., picture description) were preferred over ambiguous ones to reduce the effects of systematic bias in speech quantity in patients vs. controls[22].

References

1 Bagner DM, Melinder MRD, Barch DM. Language comprehension and working memory language comprehension and working memory deficits in patients with schizophrenia. *Schizophr Res* 2003; **60**: 299–309.
2 Morice R, McNicol D. The comprehension and production of complex syntax in schizophrenia. *Cortex* 1985; **21**: 567–80.
3 Stirling J, Hellewell J, Blakey A, Deakin W. Thought disorder in schizophrenia is associated with both executive dysfunction and circumscribed impairments in semantic function. *Psychol Med* 2006; **36**: 475–84.
4 Barattieri di San Pietro C, Barbieri E, Marelli M, de Girolamo G, Luzzatti C. Processing Argument Structure and Syntactic Complexity in People with Schizophrenia Spectrum Disorders. *J Commun Disord* 2022; **96**: 106182.
5 Condray R, Steinhauer SR, van Kammen DP, Kasparek A. The Language System in Schizophrenia: Effects of Capacity and Linguistic Structure. *Schizophrenia Bulletin* 2002; **28**: 475–90.
6 Dwyer K, David A, McCarthy R, McKenna P, Peters E. Higher-order semantic processing in formal thought disorder in schizophrenia. *Psychiatry Research* 2014; **216**: 168–76.
7 Tavano A, Sponda S, Fabbro F, *et al.* Specific linguistic and pragmatic deficits in Italian patients with schizophrenia. *Schizophrenia Research* 2008; **102**: 53–62.
8 Fraser WI, King KM, Thomas P, Kendell RE. The diagnosis of schizophrenia by language analysis. *Br J Psychiatry* 1986; **148**: 275–8.
9 Chaves MF, Rodrigues C, Ribeiro S, Mota NB, Copelli M. Grammatical impairment in schizophrenia: An exploratory study of the pronominal and sentential domains. *PLOS ONE* 2023; **18**: e0291446.
10 Özcan A, Kuruoglu G, Alptekin K, *et al.* The Production of Simple Sentence Structures in Schizophrenia. *International Journal of Arts & Sciences* 2016; **9**: 159–64.
11 Kircher TTJ, Oh TM, Brammer MJ, McGuire PK. Neural correlates of syntax production in schizophrenia. *The British Journal of Psychiatry* 2005; **186**: 209–14.
12 Schneider K, Leinweber K, Jamalabadi H, *et al.* Syntactic complexity and diversity of spontaneous speech production in schizophrenia spectrum and major depressive disorders. *Schizophr* 2023; **9**: 1–10.
13 Sanders LM, Adams J, Tager-Flusberg H, Shenton ME, Coleman M. A comparison of clinical and linguistic indices of deviance in the verbal discourse of schizophrenics. *Applied Psycholinguistics* 1995; **16**: 325–38.
14 Thomas P., Kearney G., Napier E., Ellis E., Leudar I., Johnston M. Speech and language in first onset psychosis differences between people with schizophrenia, mania, and controls. *BR J PSYCHIATRY* 1996; **168**: 337–43.
15 Morice RD, Ingram JCL. Language Analysis in Schizophrenia: Diagnostic Implications. *Aust N Z J Psychiatry* 1982; **16**: 11–21.
16 Thomas P, King K, Fraser WI. Positive and negative symptoms of schizophrenia and linguistic performance. *Acta Psychiatr Scand* 1987; **76**: 144–51.
17 Çokal D, Sevilla G, Jones WS, *et al.* The language profile of formal thought disorder. *npj Schizophrenia* 2018; **4**: 1–8.
18 Sevilla G, Rosselló J, Salvador R, *et al.* Deficits in nominal reference identify thought disordered speech in a narrative production task. *PLOS ONE* 2018; **13**: e0201545.
19 DeLisi LE. Speech disorder in schizophrenia: review of the literature and exploration of its relation to the uniquely human capacity for language. *Schizophr Bull* 2001; **27**: 481–96.
20 Shedlack K, Lee G, Sakuma M, *et al.* Language processing and memory in ill and well siblings from multiplex families affected with schizophrenia. *Schizophr Res* 1997; **25**: 43–52.
21 Tan EJ, Meyer D, Neill E, Rossell SL. Investigating the diagnostic utility of speech patterns in schizophrenia and their symptom associations. *Schizophr Res* 2021; **238**: 91–8.
22 Panikratova YaR, Vlasova RM, Akhutina TV, Tikhonov DV, Pluzhnikov IV, Kaleda VG. Executive Regulation of Speech Production in Schizophrenia: A Pilot Neuropsychological Study. *Neurosci Behav Physi* 2021; **51**: 415–22.
