# Supplementary material for: Relationship between grammar and schizophrenia: a systematic review and meta-analysis
Source: Commun Med (Lond). 2025 Jun 16;5:235. doi: 10.1038/s43856-025-00944-1 (PMC12170843; doi:10.1038/s43856-025-00944-1)
Supplement: Supplementary file 3 — Description of Additional Supplementary Files [file 43856_2025_944_MOESM3_ESM.pdf]

Legend for Supplemental Data

Supplemental Data 1: This includes links for imported databases used for literature search.
Supplemental Data 2: The PRISMA checklist to accompany the reporting procedures used in this review.
Supplemental Data 3: Table S1. List of articles excluded at the stage of data extraction.
Supplemental Data 4: Table S2. Description of the modified Newcastle-Ottawa Scale.
Supplemental Data 5: Table S3. Quality scores of the included studies.
Supplemental Data 6: Table S4. Description of the included studies.
Supplemental Data 7: Table S5. Key variables and moderators for the case-control comparisons in the meta-analysis
Supplemental Data 8: Table S6. Medications, linguistic variables and task details from the included studies.
