# Supplementary material for: Relationship between grammar and schizophrenia: a systematic review and meta-analysis
Source: Commun Med (Lond). 2025 Jun 16;5:235. doi: 10.1038/s43856-025-00944-1 (PMC12170843; doi:10.1038/s43856-025-00944-1)
Supplement: Supplementary file 4 — Supplementary Data 1 [file 43856_2025_944_MOESM4_ESM.pdf]

Supplementary Data 1: Imported Databases

All studies retrieved via the primary search are included in the following files:

File 1: [Scopus_set.ris] – Contains references from Scopus.
File 2: [webofscience_core.ris] – Contains references from Web of Science.
File 3: [psycinfo.ris] – Contains references from PsycINFO.
File 4: [pubmed-search-set2.nbib] – Contains references from PubMed.
