# Supplementary material for: Relationship between grammar and schizophrenia: a systematic review and meta-analysis
Source: Commun Med (Lond). 2025 Jun 16;5:235. doi: 10.1038/s43856-025-00944-1 (PMC12170843; doi:10.1038/s43856-025-00944-1)
Supplement: Supplementary file 5 — Supplementary Data 2 [file 43856_2025_944_MOESM5_ESM.pdf]

Supplementary Data 2: PRISMA Checklist

Section and Topic	Item #	Checklist item	Location where item is reported
**TITLE**			
Title	1	Identify the report as a systematic review.	Title: "*Syntax and Schizophrenia: A Systematic Review and Meta-Analysis of Comprehension and Production".*
**ABSTRACT**			
Abstract	2	See the PRISMA 2020 for Abstracts checklist.	Summary section: Includes objectives, methods, results, and conclusions.
**INTRODUCTION**			
Rationale	3	Describe the rationale for the review in the context of existing knowledge.	Introduction: Discusses the importance of syntax in schizophrenia and gaps in the literature.
Objectives	4	Provide an explicit statement of the objective(s) or question(s) the review addresses.	Introduction: States the aim to quantify syntactic deficits in schizophrenia and explore variability.
**METHODS**			
Eligibility criteria	5	Specify the inclusion and exclusion criteria for the review and how studies were grouped for the syntheses.	Methods, Section 2.1: Describes inclusion/exclusion criteria and grouping of syntactic domains.
Information sources	6	Specify all databases, registers, websites, organisations, reference lists and other sources searched or consulted to identify studies. Specify the date when each source was last searched or consulted.	Methods, Section 2.1: Lists databases (PubMed, PsycINFO, Scopus, Web of Science, Google Scholar).
Search strategy	7	Present the full search strategies for all databases, registers and websites, including any filters and limits used.	Supplementary Materials: Full search strategies provided.
Selection process	8	Specify the methods used to decide whether a study met the inclusion criteria of the review, including how many reviewers screened each record and each report retrieved, whether they worked independently, and if applicable, details of automation tools used in the process.	Methods, Section 2.1: Describes independent screening by two reviewers using Rayyan software.
Data collection process	9	Specify the methods used to collect data from reports, including how many reviewers collected data from each report, whether they worked independently, any processes for obtaining or confirming data from study investigators, and if applicable, details of automation tools used in the process.	Methods, Section 2.2: Describes data extraction by two independent reviewers and resolution process.
Data items	10a	List and define all outcomes for which data were sought. Specify whether all results that were compatible with each outcome domain in each study were sought (e.g. for all measures, time points, analyses), and	

Section and Topic	Item #	Checklist item	Location where item is reported
		if not, the methods used to decide which results to collect.	Methods, Section 2.2: Lists syntactic domains (comprehension, production, etc.).
	10b	List and define all other variables for which data were sought (e.g. participant and intervention characteristics, funding sources). Describe any assumptions made about any missing or unclear information.	Methods, Section 2.2: Lists demographic and clinical variables extracted.
Study risk of bias assessment	11	Specify the methods used to assess risk of bias in the included studies, including details of the tool(s) used, how many reviewers assessed each study and whether they worked independently, and if applicable, details of automation tools used in the process.	Methods, Section 2.3: Describes use of a modified Newcastle-Ottawa Scale.
Effect measures	12	Specify for each outcome the effect measure(s) (e.g. risk ratio, mean difference) used in the synthesis or presentation of results.	Methods, Section 2.4: Describes use of Cohen's d and log coefficient of variation ratio (lnCVR).
Synthesis methods	13a	Describe the processes used to decide which studies were eligible for each synthesis (e.g. tabulating the study intervention characteristics and comparing against the planned groups for each synthesis (item #5)).	Methods, Section 2.4: Describes grouping of studies by syntactic domains.
	13b	Describe any methods required to prepare the data for presentation or synthesis, such as handling of missing summary statistics, or data conversions.	Methods, Section 2.4: Describes handling of missing data and effect size calculations.
	13c	Describe any methods used to tabulate or visually display results of individual studies and syntheses.	Methods, Section 2.4: Describes use of forest plots and tables.
	13d	Describe any methods used to synthesize results and provide a rationale for the choice(s). If meta-analysis was performed, describe the model(s), method(s) to identify the presence and extent of statistical heterogeneity, and software package(s) used.	Methods, Section 2.4: Describes Bayesian meta-analysis and random-effects models.
	13e	Describe any methods used to explore possible causes of heterogeneity among study results (e.g. subgroup analysis, meta-regression).	Methods, Section 2.4: Describes meta-regression for moderators (age, study quality, etc.).
	13f	Describe any sensitivity analyses conducted to assess robustness of the synthesized results.	Methods, Section 2.4: Describes robust Bayesian meta-analysis for publication bias.
Reporting bias assessment	14	Describe any methods used to assess risk of bias due to missing results in a synthesis (arising from reporting biases).	Methods, Section 2.4: Describes robust Bayesian meta-analysis for publication bias.
Certainty assessment	15	Describe any methods used to assess certainty (or confidence) in the body of evidence for an outcome.	Methods, Section 2.4: Describes Bayesian evidence categories (e.g., weak, moderate, extreme).
**RESULTS**			

Section and Topic	Item #	Checklist item	Location where item is reported
Study selection	16a	Describe the results of the search and selection process, from the number of records identified in the search to the number of studies included in the review, ideally using a flow diagram.	Results, Section 3.1: Describes study selection process; PRISMA flow diagram in Figure 1.
	16b	Cite studies that might appear to meet the inclusion criteria, but which were excluded, and explain why they were excluded.	Supplementary Materials: Table of excluded studies with reasons.
Study characteristics	17	Cite each included study and present its characteristics.	Results, Section 3.2: Describes study characteristics.
Risk of bias in studies	18	Present assessments of risk of bias for each included study.	Supplementary Materials: Risk of bias assessments using modified Newcastle-Ottawa Scale.
Results of individual studies	19	For all outcomes, present, for each study: (a) summary statistics for each group (where appropriate) and (b) an effect estimate and its precision (e.g. confidence/credible interval), ideally using structured tables or plots.	Results, Section 3.4: Presents effect sizes and variability measures; Table 2 summarizes results.
Results of syntheses	20a	For each synthesis, briefly summarise the characteristics and risk of bias among contributing studies.	Results, Section 3.4: Summarizes study characteristics and heterogeneity.
	20b	Present results of all statistical syntheses conducted. If meta-analysis was done, present for each the summary estimate and its precision (e.g. confidence/credible interval) and measures of statistical heterogeneity. If comparing groups, describe the direction of the effect.	Results, Section 3.4: Presents meta-analysis results; Table 2 and Figures 2-4.
	20c	Present results of all investigations of possible causes of heterogeneity among study results.	Results, Section 3.4: Describes meta-regression results for moderators.
	20d	Present results of all sensitivity analyses conducted to assess the robustness of the synthesized results.	Results, Section 3.4: Describes robust Bayesian meta-analysis for publication bias.
Reporting biases	21	Present assessments of risk of bias due to missing results (arising from reporting biases) for each synthesis assessed.	Results, Section 3.4: Describes robust Bayesian meta-analysis for publication bias.
Certainty of evidence	22	Present assessments of certainty (or confidence) in the body of evidence for each outcome assessed.	Results, Section 3.4: Describes Bayesian evidence categories (e.g., weak, moderate, extreme).
**DISCUSSION**			
Discussion	23a	Provide a general interpretation of the results in the context of other evidence.	Discussion: Interprets findings in the context of prior literature.

Section and Topic	Item #	Checklist item	Location where item is reported
	23b	Discuss any limitations of the evidence included in the review.	Discussion: Highlights limitations, such as variability in study quality and sample characteristics.
	23c	Discuss any limitations of the review processes used.	Discussion: Acknowledges limitations, such as reliance on summary measures.
	23d	Discuss implications of the results for practice, policy, and future research.	Discussion: Discusses implications for interventions and future research.
**OTHER INFORMATION**			
Registration and protocol	24a	Provide registration information for the review, including register name and registration number, or state that the review was not registered.	Methods, Section 2.1: States registration on OSF (DOI: 10.17605/OSF.IO/7FZUC).
	24b	Indicate where the review protocol can be accessed, or state that a protocol was not prepared.	Methods, Section 2.1: Provides OSF link to the protocol.
	24c	Describe and explain any amendments to information provided at registration or in the protocol.	Methods, Section 2.1: Describes protocol updates.
Support	25	Describe sources of financial or non-financial support for the review, and the role of the funders or sponsors in the review.	Funding section: Lists funding sources (e.g., CIHR, Wellcome Trust).
Competing interests	26	Declare any competing interests of review authors.	Competing Interests section: L.P. reports personal fees outside the submitted work.
Availability of data, code and other materials	27	Report which of the following are publicly available and where they can be found: template data collection forms; data extracted from included studies; data used for all analyses; analytic code; any other materials used in the review.	Data Availability section: States availability of data and code on OSF.
