# Supplementary material for: Relationship between grammar and schizophrenia: a systematic review and meta-analysis
Source: Commun Med (Lond). 2025 Jun 16;5:235. doi: 10.1038/s43856-025-00944-1 (PMC12170843; doi:10.1038/s43856-025-00944-1)
Supplement: Supplementary file 6 — Supplementary Data 3 [file 43856_2025_944_MOESM6_ESM.pdf]

Table S1. List of articles excluded at the stage of data extraction.

No.	Author(s), Year	Reason for Exclusion
1	Abu-Akel, 1997	The study has a retraction notice.
2	Alqahtani et al., 2022	Focuses on non-grammatical linguistic similarities (e.g., emotions, personality traits) rather than grammar or syntax
3	Bachman-Mann, 1944	Participants included <18 years old; the control group comprised psychiatric patients; no report on syntactic variables of interest.
4	Barch & Berenbaum, 1997	The control group comprised psychiatric patients (mania)
5	Berenbaum et al., 2008	The study does not focus on grammar or syntax in speech production or comprehension.
6	Cheek & Anthony, 1970	The study lacks SDs to conduct a meta-analysis. No relevant variables reported
7	Ciampelli et al., 2023	Excluded due to the use of second-order measures only (e.g. graph-based), which involve edited or controlled verbal outputs.
8	Condray et al., 1996	This study addresses general language comprehension in relation to working memory.
9	Corcoran et al., 2018	This study focuses on at-risk youths rather than adults (under 18 years old)
10	deBoer et al., 2020	This study presents a sub-sample of an included study (repetitive)
11	Dwyer et al., 2019	The study includes an edited or controlled verbal output (confederate script technique) and lacks naturalistic speech production.
12	Ellsworth 1951	Focuses on theoretical aspects of regression rather than the empirical assessment of language production/comprehension.
13	Haas et al., 2020	Focuses on high-risk individuals rather than those with psychotic disorders; no syntactic variables of interest.
14	He et al., 2024	The study does not report variables on grammar and syntax
15	Hoffman et al., 1985	Participants included <18 years old; the control group comprised psychiatric patients; no syntactic variables of interest.
16	Hoffmann & Sledge, 1988	Control group comprised psychiatric patients
17	Jo et al., 2023	The study exclusively utilized written texts
18	Kuperberg et al., 2000	Lacks speech production/comprehension or morphosyntactic variables.
19	Lelekov, 2000	Control group comprised psychiatric patients
20	Lott et al., 2002	Control group comprised psychiatric patients
21	Morice & Ingram, 1983	This study presents the same dataset as an included study (repetitive)
22	Noel-Jorand et al., 1997	The study employed computer-assisted discourse analysis (Alceste software) ; no syntactic variables of interest.
23	Obrębska et al., 2013	This study presents the same dataset as another study (repetitive); no syntax analysis
24	Pylyshyn, 1970	The study does not have a healthy control group
25	Rossell & Batty, 2008	Focuses on semantic memory deficits - not syntactic production or comprehension.
26	Ruchsow et al., 2003	Only reaction time reported along with ERP results
27	Salome et al., 2002	No assessment/report of grammar and syntactic variables
28	Shriki et al,. 2022	Language samples were controlled or edited,
29	Silberg, 1978	Participants included <18 years old
30	Silva et al., 2023	This study presents a sub-sample of an included study (repetitive)
31	Solomon et al., 2011	Participants included <18 years old

32	Stephane et al., 2014	The study focuses on reaction time only
33	Takashima et al. 2001	No report on syntactic variables of interest
34	Tan et al., 2021	No report on syntactic variables of interest
35	Thomas et al., 1996 (2)	This study presents a sub-sample of an included study (repetitive)
36	Thomas et al., 1990	The study lacks the means and SDs to conduct a meta-analysis
37	Thomas et al., 1993	The study analyzed written language samples and controlled verbal output.
38	Voleti et al., 2019	No report on syntactic variables of interest
39	Voleti et al., 2023	This study provides only second-order derivatives (factor scores) and no means or SDs.
40	Watson et al., 2012	No report on syntactic variables of interest
41	Zhang et al., 2023	The primary linguistic criteria of syntactic complexity is unconventional in its definition of argument / adjunct clauses

References:

1. Abu-Akel A. A study of cohesive patterns and dynamic choices utilized by two schizophrenic patients in dialog, pre- and Post-Medication. *Language and Speech* 1997 Oct 1;**40**:331–51.
2. Alqahtani A, Kay ES, Hamidian S, Compton M, Diab M. A Quantitative and Qualitative Analysis of Schizophrenia Language. *arXiv* (Cornell University) 2022; published online Jan 1. https://arxiv.org/abs/2201.10430
3. Barch DM, Berenbaum H. Language generation in schizophrenia and mania: the relationships among verbosity, syntactic complexity, and pausing. *Journal of Psycholinguistic Research* 1997; published online Jan 1.**26**:401–12.DOI: 10.1023/a:1025026019107
4. Berenbaum H, Kerns JG, Vernon LL, Gomez JJ. Cognitive correlates of schizophrenia signs and symptoms: I. verbal communication disturbances. *Psychiatry Research* 2008; published online Apr 19;**159**(1–2):147–56.DOI: 10.1016/j.psychres.2007.08.016
5. Cheek FE, Anthony R. Personal Pronoun Usage in Families of Schizophrenics And Social Space Utilization. *Family Process* 1970 Dec;**9**(4):431–47.
6. Ciampelli S, De Boer JN, Voppel AE, Corona Hernandez H, Brederoo SG, Van Dellen E, et al. Syntactic Network Analysis in Schizophrenia-Spectrum Disorders. *Schizophrenia Bulletin* 2023 Mar 22;**49**:S172–82.
7. Condray R, Steinhauer SR, Van Kammen DP, Kasparek A. Working memory capacity predicts language comprehension in schizophrenic patients. *Schizophrenia Research* 1996 May;**20**:1–13.
8. Corcoran CM, Carrillo F, Fernández-Slezak D, Bedi G, Klim C, Javitt DC, et al. Prediction of psychosis across protocols and risk cohorts using automated language analysis. *World Psychiatry* 2018 Feb;**17**(1):67–75.
9. De Boer JN, Van Hoogdalem M, Mandl RCW, Brummelman J, Voppel AE, Begemann MJH, et al. Language in schizophrenia: relation with diagnosis, symptomatology and white matter tracts. *npj Schizophr* 2020 Apr 20;**6**:10.
10. Dwyer K, David AS, McCarthy R, McKenna P, Peters E. Linguistic alignment and theory of mind impairments in schizophrenia patients' dialogic interactions. *Psychol Med* 2020 Oct;**50**(13):2194–202.
11. Ellsworth RB. The regression of schizophrenic language. *Journal of Consulting Psychology* 1951 Oct;**15**(5):387–91.
12. Haas SS, Doucet GE, Garg S, Herrera SN, Sarac C, Bilgrami ZR, et al. Linking language features to clinical symptoms and multimodal imaging in individuals at clinical high risk for psychosis. *Eur Psychiatr* 2020;**63**(1):e72.
13. He R, Palominos C, Zhang H, Alonso-Sánchez MF, Palaniyappan L, Hinzen W. Navigating the semantic space: Unraveling the structure of meaning in psychosis using different computational language models. *Psychiatry Research* 2024 Mar;**333**:115752.
14. Hoffman RE, Hogben GL, Smith H, Calhoun WMF. Message disruptions during syntactic processing in schizophrenia. *Journal of Communication Disorders* 1985 Jun;**18**(3):183–202.
15. Hoffman RE, Sledge W. An analysis of grammatical deviance occurring in spontaneous schizophrenic speech. *Journal of Neurolinguistics* 1988 Jan;**3**(1):89–101.
16. Jo YT, Park SY, Park J, Lee J, Joo YH. Linguistic anomalies in the language of patients with schizophrenia. *Schizophrenia Research: Cognition* 2023 Mar;**31**:100273.

17. Kuperberg GR, McGUIRE PK, David AS. Sensitivity to linguistic anomalies in spoken sentences: a case study approach to understanding thought disorder in schizophrenia. *Psychol Med* 2000 Mar;**30**(2):345–57.
18. Lelekov T, Franck N, Dominey PF, Georgieff N. Cognitive sequence processing and syntactic comprehension in schizophrenia. *NeuroReport* 2000 Jul;**11**(10):2145–9.
19. Lott PR, Guggenbühl S, Schneeberger A, Pulver AE, Stassen HH. Linguistic Analysis of the Speech Output of Schizophrenic, Bipolar, and Depressive Patients. *Psychopathology*. 2002;**35**(4):220–7.
20. Mann MB. III. The quantitative differentiation of samples of written language. *The Psychological Monographs* 1944 Jan 1;**56**(2):39–74.
21. Morice RD, Ingram JCL. Language complexity and age of onset of schizophrenia. *Psychiatry Research* 1983 Jul;**9**(3):233–42.
22. Noeljorand M, Reinert M, Giudicelli S, Dassa D. A new approach to discourse analysis in psychiatry, applied to a schizophrenic patient's speech. *Schizophrenia Research* 1997 Jun 20;**25**(3):183–98.
23. Obrębska M. Frequency Analysis of Singular First-Person Pronouns and Verbs in the Utterances of Schizophrenia Patients and Healthy Controls. A Research Report. *Lingua Posnaniensis* 2013 Jun 1;**55**(1):87–98.
24. Pylyshyn ZW. CLINICAL CORRELATES OF SOME SYNTACTIC FEATURES OF PATIENTS' SPEECH. *The Journal of Nervous and Mental Disease* 1970 Apr 1;**150**(4):307–16.
25. Rossell S, Batty R. Elucidating semantic disorganisation from a word comprehension task: Do patients with schizophrenia and bipolar disorder show differential processing of nouns, verbs, and adjectives? *Schizophrenia Research* 2008 Jul;**102**(1–3):63–8.
26. Ruchsow M, Trippel N, Groen G, Spitzer M, Kiefer M. Semantic and syntactic processes during sentence comprehension in patients with schizophrenia: evidence from event-related potentials. *Schizophrenia Research* 2003 Nov;**64**(2–3):147–56.
27. Salomé F, Boyer P, Fayol M. Written but not oral verbal production is preserved in young schizophrenic patients. *Psychiatry Research* 2002 Aug;**111**(2–3):137–45.
28. Shriki Y, Ziv I, Dershowitz N, Harel E, Bar K. Masking Morphosyntactic Categories to Evaluate Salience for Schizophrenia Diagnosis. In: Proceedings of the Eighth Workshop on Computational Linguistics and Clinical Psychology. Seattle, USA: *Association for Computational Linguistic*s 2022 [cited 2024 Oct 19]. p. 148–57. Available from: https://aclanthology.org/2022.clpsych-1.13
29. Silberg JL. The development of pronoun usage in the psychotic child. *J Autism Dev Disord* 1978 Dec;**8**(4):413–25.
30. Silva AM, Limongi R, MacKinley M, Ford SD, Alonso-Sánchez MF, Palaniyappan L. Syntactic complexity of spoken language in the diagnosis of schizophrenia: A probabilistic Bayes network model. *Schizophrenia Research* 2023 Sep;**259**:88–96.
31. Solomon M, Olsen E, Niendam T, Ragland JD, Yoon J, Minzenberg M, et al. From lumping to splitting and back again: Atypical social and language development in individuals with clinical-high-risk for psychosis, first-episode schizophrenia, and autism spectrum disorders. *Schizophrenia Research* 2011 Sep;**131**(1–3):146–51.
32. Stephane M, Kuskowski M, Gundel J. Abnormal dynamics of language in schizophrenia. *Psychiatry Research* 2014 May;**216**(3):320–4.
33. Takashima A, Ohta K, Matsushima E, Toru M. The event-related potentials elicited by content and function words during the reading of sentences by patients with schizophrenia. *Psychiatry Clin Neurosci* 2001 Dec;**55**(6):611–8.
34. Tan EJ, Meyer D, Neill E, Rossell SL. Investigating the diagnostic utility of speech patterns in schizophrenia and their symptom associations. *Schizophrenia Research* 2021 Dec;**238**:91–8.
35. Thomas P, King K, Fraser WI, Kendell RE. Linguistic Performance in Schizophrenia: a Comparison of Acute and Chronic Patients. *Br J Psychiatry* 1990 Feb;**156**(2):204–10.
36. Thomas P, Leudar I, Newby D, Johnston M. Syntactic processing and written language output in first onset psychosis. *Journal of Communication Disorders* 1993 Dec;**26**(4):209–30.
37. Thomas P. Syntactic Complexity and Negative Symptoms in First Onset Schizophrenia. *Cognitive Neuropsychiatry* 1996 Aug;**1**(3):191–200.
38. Voleti R, Woolridge S, Liss JM, Milanovic M, Bowie CR, Berisha V. Objective Assessment of Social Skills Using Automated Language Analysis for Identification of Schizophrenia and Bipolar Disorder. *ISCA* 2019 [cited 2024 Oct 19]. p. 1433–7. Available from: https://www.isca-archive.org/interspeech_2019/voleti19_interspeech.html
39. Voleti R, Woolridge SM, Liss JM, Milanovic M, Stegmann G, Hahn S, et al. Language Analytics for Assessment of Mental Health Status and Functional Competency. *Schizophrenia Bulletin* 2023 Mar 22;**49**(Supplement_2):S183–95.
40. Watson AR, Defteralı Ç, Bak TH, Sorace A, McIntosh AM, Owens DGC, et al. Use of second-person pronouns and schizophrenia. *Br J Psychiatry* 2012 Apr;**200**(4):342–3.
41. Zhang H, Parola A, Zhou Y, Wang H, Bliksted V, Fusaroli R, et al. Linguistic markers of psychosis in Mandarin Chinese: Relations to theory of mind. *Psychiatry Research* 2023 Jul;**325**:115253.
