# Supplementary material for: Relationship between grammar and schizophrenia: a systematic review and meta-analysis
Source: Commun Med (Lond). 2025 Jun 16;5:235. doi: 10.1038/s43856-025-00944-1 (PMC12170843; doi:10.1038/s43856-025-00944-1)
Supplement: Supplementary file 7 — supplementary data 4 [file 43856_2025_944_MOESM7_ESM.pdf]

Table S2. Description of the modified Newcastle–Ottawa Scale.

**Quality Indicator**	**Description**	**Scoring Criteria (0-2 points)**
1. Case Definition	Is the psychosis diagnosis (e.g., SZ) clearly defined using standard criteria (DSM-5, ICD)?	2: Standard diagnostic criteria used (e.g., DSM-5/DSM/RDC).<br>1: Self-report or unclear.<br>0: No definition.
2. Control Group	Are controls well-defined and matched (e.g., similar demographic characteristics - age, sex, education, and language profile)?	2: Matched on age/sex, education and language profile<br>1: Matched on less than 2 aspects<br>0: Not adequate matching (1 or no matched features)
3. Outcome Assessment (Language)	Are validated methods used to measure language comprehension/production?	2: Validated rater-scored tests or replicable automated approaches used.<br>1: Custom or non-validated tests.<br>0: No description.
4. Data provided	Are appropriate summary measures (SD and mean for each group for each variable of interest) reported?	2: Reported explicitly<br>1: Exact numbers obtained from raw data made available<br>0: Not reported - approximate numbers obtained from figures
5. Representativeness of Patients	Are the cases representative of the wider population (e.g., all eligible cases included)?	2: All eligible cases included.<br>1: Partial inclusion: only hospitalized patients or only those with specific symptoms selected (e.g. FTD+ only, or negative symptoms only)<br>0: No clear description.
6. Language Variable Reported	Do the measured variables match with the categories described?	2: Reported variables match fully with extracted categories.<br>1: Overlaps with other categories (e.g. reporting all pronouns instead of first person alone)<br>0: Only partial match as variables not fully defined (e.g. reporting complexity as a variable without details on how it was ascertained)
