# Supplementary material for: Relationship between grammar and schizophrenia: a systematic review and meta-analysis
Source: Commun Med (Lond). 2025 Jun 16;5:235. doi: 10.1038/s43856-025-00944-1 (PMC12170843; doi:10.1038/s43856-025-00944-1)
Supplement: Supplementary file 8 — Supplementary Data 5 [file 43856_2025_944_MOESM8_ESM.pdf]

Table S3. Quality scores of the included studies.

	Case definition	Control ascertainment	Exposure assessment	Data provided	Sample representativeness	Reported variables	Total score
**Anand et al. 1994**	2	2	1	2	1	2	10
**Arslan et al. 2024**	2	1	2	2	1	2	10
**Bagner et al. 2003**	2	2	1	2	1	1	9
**Barattieri di San Pietro et al. 2022**	2	2	2	2	1	2	11
**Barrera et al. 2005**	2	1	2	2	1	2	10
**Buck & Penn 2015**	2	1	2	2	1	2	10
**Çabuk et al. 2024**	2	2	2	2	1	2	11
**Chaves et al. 2023**	2	1	1	2	2	1	9
**Çokal et al. 2018**	2	2	2	2	1	2	11
**Çokal et al. 2019**	2	1	1	2	1	2	9
**Condray et al. 1995**	2	2	2	2	1	2	11
**Condray et al. 2002**	2	2	2	2	1	1	10
**Dalal et al. 2024**	2	1	2	1	1	2	9
**de Boer et al. 2021**	2	2	2	1	1	2	10
**DeLisi 2001**	2	1	1	2	1	1	8
**Delvecchio et al. 2019**	2	1	2	2	1	2	10
**Dwyer et al. 2014**	2	2	1	1	1	2	9
**Fraser et al. 1986**	2	2	2	2	1	2	11
**Gargano et al. 2022**	2	1	1	2	1	2	9
**King et al. 1990**	1	1	2	2	1	2	9
**Kircher et al. 2005**	2	2	1	2	1	2	10
**Kuperberg et al. 2006 (1)**	2	2	1	1	1	2	9
**Kuperberg et al. 2006 (2)**	2	1	1	2	0	2	8
**Lee et al. 2016**	2	2	1	2	1	2	10
**Li 2024**	2	2	1	1	1	2	9
**Liang et al. 2022**	2	1	2	2	1	2	10
**Morgan et al. 2021**	2	1	2	1	1	2	9

**Morice & Ingram 1982**	**2**	**1**	**2**	**2**	**1**	**2**	**10**
**Morice and McNicol 1985**	**2**	**1**	**2**	**2**	**1**	**2**	**10**
**Moro et al. 2015**	**2**	**2**	**2**	**2**	**1**	**2**	**11**
**Özcan et al. 2017**	**2**	**2**	**2**	**2**	**1**	**2**	**11**
**Panikratova et al. 2021**	**2**	**1**	**1**	**2**	**1**	**2**	**9**
**Perlini et al. 2012**	**2**	**2**	**2**	**1**	**2**	**2**	**11**
**Sanders et al. 1995**	**2**	**1**	**1**	**2**	**1**	**1**	**8**
**Schneider et al. 2023**	**2**	**1**	**2**	**2**	**1**	**2**	**10**
**Sevilla et al. 2018**	**2**	**1**	**1**	**2**	**1**	**2**	**9**
**Shedlack et al. 1997**	**2**	**2**	**1**	**2**	**1**	**1**	**9**
**Stephane et al. 2007**	**2**	**2**	**1**	**2**	**1**	**2**	**10**
**Stirling et al. 2006**	**2**	**1**	**2**	**2**	**2**	**2**	**11**
**Tan et al. 2016**	**2**	**2**	**2**	**1**	**1**	**2**	**10**
**Tang et al. 2021**	**2**	**1**	**2**	**2**	**1**	**2**	**10**
**Tavano et al. 2008**	**2**	**2**	**2**	**2**	**1**	**2**	**11**
**Thomas et al. 1987**	**2**	**1**	**2**	**1**	**1**	**2**	**9**
**Thomas et al. 1996 (1)**	**2**	**1**	**2**	**2**	**1**	**2**	**10**
**Vogel et al. 2009**	**2**	**2**	**2**	**2**	**1**	**2**	**11**
