# Supplementary material for: Relationship between grammar and schizophrenia: a systematic review and meta-analysis
Source: Commun Med (Lond). 2025 Jun 16;5:235. doi: 10.1038/s43856-025-00944-1 (PMC12170843; doi:10.1038/s43856-025-00944-1)
Supplement: Supplementary file 9 — Supplementary Data 6 [file 43856_2025_944_MOESM9_ESM.pdf]

Table S4. Description of the included studies.

Author(s), Year	n(pts/ctrls)	Mean age/SD pts.	Diagnosis	Stage of illness	Language of assessment / Country	Proportion medicated	FTD Status	Symptom Severity (Scale/Severity Index Score)	Variables Extracted for Meta-analysis[92]
Anand et al. 1994	24/24	23.9 (5.4)	DSM-III-R Schizophreniform, Schizophrenia, Schizoaffective disorder	Established	English/ Australia	~100%	No split	N/A	Error Detection (C)
Arslan et al. 2024	53/50	22.96 (8.05)	DSM-IV psychosis other than bipolar disorder	First Episode	Turkish/ Turkey	100%	No split	N/A	Production Length (P)
Bagner et al. 2003	27/28	38.81 (9.72)	DSM-IV Schizophrenia	Established	English/USA	89%	No split	PANSS: 59.8/ 0.165	Syntax Comprehension (C)
Barattieri di San Pietro et al. 2022	34/34	48.82 (10.4)	DSM-5 Schizophreniform, Schizophrenia, Schizoaffective, and other psychotic disorder	Established	Italian/Italy	100%	No split	BPRS-E: 44.29/0.141	Syntax Comprehension (C)
Barrera et al. 2005	31/17	FTD: 47.1 (7.9), non-FTD: 41.2 (9.2)	RDC Schizophrenia	Established	English/UK	100%	FTD+nFTD	N/A	Syntax Comprehension (C)
Buck & Penn 2015	42/48	N/A	DSM-IV Schizophrenia or schizoaffective disorder	Established	English/USA	N/A	No split	N/A	Production Length (P)
Çabuk et al. 2024	38/38	38.82 (8.16)	DSM-5 Schizophrenia	Established	Turkish/ Turkey	100%	No split	N/A	Production Length (P)

Chaves et al. 2023	31/40	34.79 (9.60)	DSM-IV Schizophrenia	First Episode & Established	Brazilian Portuguese/ Brazil	100% both samples	No split	PANSS: SCZ: 69/ 0.216 FEP: 69.27/0.22	Global Complexity (P)
Çokal et al. 2018	30/15	50 (14.6) (SZ + FTD), 38 (7.3) (SZ−FTD)	DSM-IV Schizophrenia	Established	English/UK	100%	FTD+nFTD	PANSS: FTD: 88/0.32 nFTD: 74/0.24	Phrasal Complexity (P), Production Integrity (P)
Çokal et al. 2019	25/13	50 (14.6) (SZ + FTD), 38 (7.3) (SZ−FTD)	DSM-IV Schizophrenia	Established	English/UK	N/A	FTD+nFTD	PANSS: FTD: 113.25/0.46 nFTD: 86.00/0.31	Syntax Comprehension (C)
Condray et al. 1995	15/15	36.2	DSM-III-R Schizophrenia/schizoaffective	Established	English/USA	100% (on/off phase)	No split	N/A	Syntax Comprehension (C)
Condray et al. 2002	32/22	42.4 (7.9)	DSM-III-R Schizophrenia	Established	English/USA	90.60%	No split	N/A	Syntax Comprehension (C)
Dalal et al. 2024	90/39	FEP: 22.24 (4.37); SCZ: 28.47 (7.64)	DSM-5 Schizophrenia, Schizoaffective, Schizophreniform, psychosis NoS, affective psychosis	First Episode & Established	English/ Canada	51.4% FES and 100% of non-FES	No split	PANSS-8: FEP: 24.41/0.34 SCZ: 14.12/0.15	Global Complexity (P), Phrasal Complexity (P),
de Boer et al. 2021	41/40	28.41	DSM-IV Schizophrenia	Established	Dutch/ Netherlands	100%	No split	PANSS: 52/0.12	Production Length (P), Phrasal Complexity (P)
DeLisi 2001	38/12	Chronic: 33.8 (8) years, First episode: 23.4 (5)	DSM-IV Schizophrenia or Schizophreniform disorder	First Episode & Established	English/USA	100%	No split	N/A	Phrasal Complexity (P), Production Integrity (P)

Delvecchio et al. 2019	166/ 106	30.5 (10.01)	ICD-10 Schizophrenia (64), Schizotypal disorder (4), Delusional disorder (33), Brief psychotic disorder (33), Schizoaffective disorder (20), Psychosis NoS (12).	First Episode	Italian/Italy	54.6%	No split	PANSS: 67.2/0.21	Syntax Comprehension (C)
Dwyer 2014	32/1 5	FTD patients: 41 (12.6) Non-FTD patients: 37.5 (10.2)	DSM-IV Schizophrenia	Established	English/UK	100%	FTD+nFTD	N/A	Syntax Comprehension (C)
Fraser et al. 1986	50/5 0	28.1 (8.98)	RDC Schizophrenia	Established	English/UK	70%	No split	N/A	Phrasal Complexity (P), Production Integrity (P)
Gargano et al. 2022	133/ 133	28.93 (9.05)	DSM-IV First Episode Psychosis	First Episode	Italian/Italy	N/A	No split	PANSS: 36.2/0.034	Production Length (P), Production Integrity (P)
King et al. 1990	11/9	24.7 (5.8)	RDC Schizophrenia	Established	English/N/A	N/A	No split	N/A	Production Length (P)
Kircher et al. 2005	6/6	N/A	DSM–IV Schizophrenia	Established	English/N/A	100%	FTD only	N/A	Global Complexity (P)
Kuperberg et al. 2006 (1)	20/2 0	42 (9)	DSM-IV Schizophrenia	Established	English/UK & USA	100%	No split	PANSS: 66.4/0.20	Error Detection (C)
Kuperberg et al. 2006 (2)	20/2 0	43 (10)	DSM-IV Schizophrenia	Established	English/USA	100%	No split	PANSS: 59.8/0.166	Error Detection (C)
Lee et al. 2016	26	33.9 (7.2)	DSM-IV Schizophrenia	Established	Korean/South Korea	100%	No split	BPRS: 24.5/0.06	Error Detection (C)

Li et al. 2024	38/25	37.58 (8.40)	DSM-5 Schizophrenia	Established	Chinese/China	N/A	No split	PANSS: 64.68/0.19	Production Length (P), Phrasal Complexity (P)
Liang et al. 2022	66/36	22.82 (4.77)	DSM-5 Schizophrenia spectrum (nonaffective psychosis)	First Episode	English/Canada	58%	No split	PANSS-8: 25.2/0.36	Production Length (P)
Morgan et al. 2021	16/13	N/A	DSM-IV or ICD-10 criteria for Schizophrenia and other psychotic disorders	First Episode	English/South London	37.50%	No split	N/A	Production Length (P)
Morice & Ingram 1982	34/18	26.7 (6.3)	ICD-8 Schizophrenia and Mania ICD-8	Established	English/ Australia	100%	No split	N/A	Global Complexity (P), Production Integrity (P)
Morice and McNicol 1985	17/19	30	DSM-III Schizophrenia	Established	English/ Australia	100%	No split	N/A	Syntax Comprehension (C), Global Complexity (P), Phrasal Complexity (P)
Moro et al. 2015	58/30	34.72 (8.23)	DSM-IV-TR Schizophrenia, treatment-responsive	Established	Italian/Italy	100%	No split	PANSS: 67.9/0.21	Error Detection (C)
Özcan et al. 2017	50/50	41.98 (4.57)	DSM-IV Schizophrenia	Established	Turkish/Turkey	100%	No split	N/A	Global Complexity (P)
Panikratova et al. 2021	25/27	30.3 (13.2)	ICD-10 Schizophrenia	Established	Russian/Russia	N/A	No split	PANSS: 72.4/0.236	Production Length (P), Phrasal Complexity (P)
Perlini et al. 2012	30/30	39.70 (10.88)	DSM-IV Schizophrenia	Established	Italian/Italy	96.70%	No split	BPRS: 42/0.22	Syntax Comprehension (C), Production Length (P), Production Integrity (P)
Sanders et al. 1995	11/11	N/A	DSM-III Schizophrenia	Established	English/N/A	N/A	No split	N/A	Production Length (P), Global

									Complexity (P), Clausal Sophist
Schneider et al. 2023	34/40	42.47 (13.11)	DSM-IV-TR Schizophrenia Spectrum Disorders	Established	German/ Germany	100%	No split	SANS+SAPS (no positive FTD/ attention):31.36/ 0.13	Production Length (P), Global Complexity (P), Phrasal Complexity (P)
Sevilla et al. 2018	40/14	SZ+TD: 41.21 (12.48); SZ-TD: 41.35 (8.99)	DSM-IV Schizophrenia with or without Thought Disorder (TD)	Established	Spanish/Spain	100%	FTD+nFTD	PANSS: 75.98/0.255 nFTD: 66.55/0.20 FTD: 85.40/0.31	Phrasal Complexity (P)
Shedlack et al. 1997	37/17	33.8 (7.7)	DSM-III-R Schizophrenia, Schizoaffective, Schizotypal disorders and Psychosis NoS	Established	English/USA	76.50%	No split	N/A	Phrasal Complexity (P), Production Integrity (P)
Stephane et al. 2007	22/11	51 (7)	DSM-IV Schizophrenia or schizoaffective disorder	Established	English/USA	95.50%	No split	BPRS: 44/0.24	Error Detection (C)
Stirling et al. 2006	30/18	34.33 (10.39)	DSM-IV Schizophrenia	Established	English/N/A	93%	Examined post-hoc	N/A	Syntax Comprehension (C)
Tan et al. 2016	57/48	43.40 (10.85)	DSM-IV Schizophrenia/ Schizoaffective disorder	Established	English/ Australia	100%	No split	PANSS: 59.3/0.163	Syntax Comprehension (C)
Tang et al. 2021	20/11	36.5 (7.2)	DSM-IV Schizophrenia	Established	English/USA	N/A	No split	N/A	Production Length (P)
Tavano et al. 2008	37/37	39.73 (13.05)	DSM-IV Schizophrenia	Established	Italian/Italy	100%	No split	N/A	Syntax Comprehension (C), Production Length (P), Global Complexity (P)

Thomas et al. 1987	18/10	24.8 (4.4)	RDC Schizophrenia	Established	English/ Scotland	N/A	No split	N/A	Production Length (P), Global Complexity (P), Phrasal Complexity (P), Production Integrity (P)
Thomas et al. 1996	38/16	26.96	RDC Schizophrenia	Established	English/UK	100%	No split	N/A	Production Length (P), Global Complexity (P), Phrasal Complexity (P), Production Integrity (P)
Vogel et al. 2009	15/12	N/A	DSM-IV Schizophrenia	Established	English/ Australia	100%	No split	N/A	Production Integrity (P)

NOTES: FTD=Formal Thought Disorder. nFTD=no-Formal Thought Disorder. FDRs=First Degree Relatives. DSM=Diagnostic and Statistical Manual of Mental Disorders. RDC=Research Diagnostic Criteria. ICD=International Classification of Diseases. PANSS=Positive and Negative Syndrome Scale. SANS=Scale for the Assessment of Negative Symptoms. SAPS=Scale for the Assessment of Positive Symptoms. PSE=Present State Examination. FEP=First Episode Psychosis. P= Production. C= Comprehension.
