# Supplementary material for: Relationship between grammar and schizophrenia: a systematic review and meta-analysis
Source: Commun Med (Lond). 2025 Jun 16;5:235. doi: 10.1038/s43856-025-00944-1 (PMC12170843; doi:10.1038/s43856-025-00944-1)
Supplement: Supplementary file 10 — Supplementary Data 7 [file 43856_2025_944_MOESM10_ESM.pdf]

Table S5. Key variables and moderators for the case-control comparisons in the meta-analysis

Authors	Variables	n(pts/ ctrls)	Mean/SD pts.	Mean/SD ctrls.	Effect Size (d)	Standard Error (SE)	Mean age pts.	Mean age ctrls.	%females pts.	CPZ dose mean	English	Quality Score	Speech task
Bagner et al. 2003	SC: Understanding long/object-relative clauses (Q&A)	27/28	0.66 (0.185)	0.825 (0.135)	1.02	0.29	38.81	35.39	33.33	N/A	Yes	9	N/A
Barrera et al. 2005 (FTD)	SC: Understanding complex sentences (SPM)	15/17	75.4 (3.9)	78.5 (1.6)	1.044	0.38	47.1	41	22.58	N/A	Yes	10	N/A
Barrera et al. 2005 (nFTD)	SC: Understanding complex sentences (SPM)	16/17	77.8 (2.3)	78.5 (1.6)	0.35	0.35	41.2	41	22.58	N/A	Yes	10	N/A
Çokal et al. 2019 (FTD)	SC: Understanding complex sentences (SPM)	12/13	0.71 (0.201)	0.97 (0.038)	1.80	0.47	47.92	45.31	16.7	N/A	Yes	9	N/A
Çokal et al. 2019 (nFTD)	SC: Understanding complex sentences (SPM)	13/13	0.92 (0.068)	0.97 (0.038)	0.90	0.41	47.92	45.31	31	N/A	Yes	9	N/A
Condray et al. 1995	SC: Understanding syntax-based relational concepts (R2 Luria-Nebraska Q&A - errors)	15/15	1.8 (1.64)	0.2 (0.56)	1.31	0.40	36.2	35.2	0	243	Yes	11	N/A
Condray et al. 2002	SC: Understanding objects/actors in embedded clauses (Q&A)	32/22	0.63 (0.26)	0.8 (0.19)	0.75	0.29	42.4	38.9	0	504	Yes	10	N/A
Delvecchio et al. 2019	SC: Understanding complex sentences (SPM)	166/106	2.9 (2.8)	1.1 (1.4)	0.81	0.13	30.5	31.8	45.87	252	No	10	N/A
Barattieri di San Pietro et al. 2022	SC: Understanding object-relative complex sentences (SPM)	34/34	0.624 (0.486)	0.918 (0.276)	0.74	0.25	48.82	48.23	26.47	N/A	No	11	N/A
Dwyer et al. 2014 (FTD)	SC: Sentence pairs task judgment (Neutral)	14/15	58.55 (20.59)	84.37 (13.09)	1.50	0.42	41	35.9	28.6	N/A	Yes	9	N/A
Dwyer et al. 2014 (nFTD)	SC: Sentence pairs task judgment (Neutral)	18/15	74.28 (16.09)	84.37 (13.09)	0.69	0.36	37.5	35.9	22.2	N/A	Yes	9	N/A
Morice & McNicol 1985	SC: Understanding complex part of Token Test (instructions)	17/19	16.8 (5.2)	23.4 (2.5)	1.62	0.38	30	30	29	N/A	Yes	10	N/A
Perlini et al. 2012	SC: Understanding complex sentences (SPM - errors) using TCGB	27/28	4.59 (3.94)	1.11 (1.13)	1.20	0.29	39.7	38.53	20	354	No	11	N/A

Stirling et al. 2006	SC: Understanding complex sentences (SPM) in TROG	30/18	17.6 (1.25)	19.67 (0.67)	2.06	0.37	34.33	36.22	40	573	Yes	11	N/A
Tan et al. 2016	SC: Understanding changed syntax in pairs (Q&A - errors)	57/48	4.47 (2.56)	2.33 (1.62)	1.00	0.21	43.4	39.83	47.4	479	Yes	11	N/A
Tavano et al. 2008	SC: Understanding complex sentences (SPM - errors)	37/37	5.37 (5.24)	0.88 (1.67)	1.15	0.25	39.73	38.16	70.27	N/A	No	11	N/A
Anand et al. 1994	ED: Error rate for incorrect sentences	24/24	31.1 (14)	17.9 (10.2)	1.08	0.31	23.9	24.8	25	319	Yes	10	N/A
Kuperberg et al. 2006 (1)	ED: Error rate for incorrect sentences	20/20	11.35 (9.08)	9.41 (21.14)	0.12	0.32	41	42	15	410	Yes	9	N/A
Kuperberg et al. 2006 (2)	ED: Error rate for incorrect sentences	20/20	42.67 (35.61)	10.61 (19.13)	1.12	0.34	41	43	20	467	Yes	8	N/A
Stephane et al. 2007	ED: Accuracy rate for incorrect sentences	22/11	0.71 (0.188)	0.88 (0.16)	0.97	0.39	51	47	9	308	Yes	9	N/A
Moro et al. 2015	ED: Accuracy rate for incorrect sentences	58/30	70.91 (20.23)	89.18 (8.06)	1.19	0.24	34.72	37.93	44.82	250	No	10	N/A
Lee et al. 2016	ED: Accuracy rate for incorrect sentences	26/29	87.4 (14.94)	98.42 (3.32)	1.02	0.29	33.9	33.6	38.5	654	No	10	N/A
King et al. 1990 (FUP)	PL: MLU	11/9	9.43 (2.37)	11.1 (1.8)	0.79	0.47	24.7	33.1	30	N/A	Yes	9	Free speech
King et al. 1990 (Original)	PL: MLU	51/50	9.38 (2.22)	10.68 (1.79)	0.64	0.21	28.1	38.2	30	N/A	Yes	9	Free speech
Sanders et al. 1995	PL: MLU	11/11	6.24 (1.7)	8.01 (2)	0.95	0.45	N/A	N/A	N/A	N/A	Yes	8	Free speech
Thomas et al. 1996	PL: MLU	38/16	7.38 (1.78)	8.73 (1.62)	0.79	0.31	26.96	26.96	36	N/A	Yes	10	Free speech
Tavano et al. 2008	PL: MLU	37/37	6.21 (1.47)	5.71 (0.82)	0.42	0.24	39.73	38.16	29.7	N/A	No	11	Picture description
Perlini et al. 2012	PL: MLU	30/30	4.49 (0.93)	5.63 (1.28)	1.02	0.27	39.7	38.53	20	354	No	11	Picture description
Panikratova et al. 2021	PL: MLS	25/27	6.4 (1.9)	8.3 (2.67)	0.82	0.29	30.3	26.1	0	N/A	No	9	Both
deBoer et al. 2021	PL: MLU	41/40	14.89 (6.84)	19.1 (7.72)	0.58	0.23	28.41	31.7	24.4	423	No	10	Free speech
Gargano et al. 2022	PL: MLU	133/133	6.23 (1.53)	7.19 (1.67)	0.60	0.13	28.93	33.07	39.9	N/A	No	9	Picture description

Liang et al. 2022	PL: MLS	66/13	14.37 (4.58)	14.21 (2.74)	0.040	0.21	22.82	21.53	18.2	150	Yes	10	Picture description
Morgan et al. 2021	PL: MLS	16/13	11.2283 (4.435)	17.1983 (4.54)	1.330	0.410	24.5	26.5	18.7	N/A	Yes	8	Both
Schneider et al. 2023	PL: MLU	34/40	13.8 (3.74)	17.91 (4.18)	1.04	0.25	42.47	40.83	10	403	No	10	Free speech
Arslan et al. 2024	PL: MLS	53	4.67 (0.95)	6.35 (1.1)	1.63	0.23	22.96	22.98	45.3	222	No	10	Picture description
Li et al. 2024	PL: MLU	38	27.57 (12.4)	56.1 (38.2)	1.00	0.27	37.58	37.07	8	286	No	9	Free speech
Çabuk et al. 2024	PL: MLS	38	4.681 (1.492)	6.571 (1.684)	1.19	0.25	38.82	37.97	24.7	504	No	11	Free speech
Buck & Penn 2015	PL: MLS	42	13.270 (4.310)	21.900 (8.620)	1.27	0.23	N/A	N/A	N/A	N/A	Yes	10	Free speech
Tang et al. 2021	PL: MLS	20	17.5 (3.1)	14.4 (4.3)	0.82	0.39	36.5	35.6	45	N/A	Yes	10	Free speech
Morice & McNicol 1985	PC: Depth of embedding	17/19	1.29 (0.12)	1.45 (0.14)	1.23	0.36	30	30	29	N/A	Yes	10	Free speech
Fraser et al. 1986a	PC: Depth of embedding	50/50	1.3 (0.15)	1.39 (0.15)	0.60	0.20	28.1	38.2	30	N/A	Yes	10	Both
Sanders et al. 1995	PC: Number of clauses in complex sentences	11/11	2.5 (0.29)	2.5 (0.21)	0	0.43	N/A	N/A	N/A	N/A	Yes	8	Free speech
Thomas et al. 1996	PC: Depth of embedding	38/16	1.09 (0.35)	1.34 (0.09)	0.98	0.31	26.96	26.96	36	N/A	Yes	10	Free speech
Shedlak et al. 1997a	PC: Complement clauses	37/17	1.55 (1.75)	1.95 (0.55)	0.31	0.29	33.8	31.9	21.6	N/A	Yes	9	Free speech
DeLisi 2001 (FEP)	PC: Number of conjoined clauses	9/12	29.9 (24)	45.8 (27)	0.62	0.45	23.4	32.6	33.3	N/A	Yes	8	Both
DeLisi 2001 (SCZ)	PC: Number of conjoined clauses	29/12	20.2 (16)	45.8 (27)	1.15	0.37	33.8	32.6	24.1	N/A	Yes	8	Both
Çokal et al. 2018 (FTD)	PC: Embedded clauses/ utterance	15/15	0.23 (0.13)	0.47 (0.13)	1.74	0.43	50	45	13.3	N/A	Yes	10	Picture description
Çokal et al. 2018 (nFTD)	PC: Embedded clauses/ utterance	15/15	0.37 (0.15)	0.47 (0.13)	0.69	0.38	38	45	33.3	N/A	Yes	10	Picture description
Sevilla et al. 2018	PC: Complement clauses	40/14	1.5 (1.93)	1.93 (1.98)	0.22	0.31	41.28	39.6	40	848	Yes	9	Free speech
Panikratova et al. 2021	PC: Number of clauses in complex sentences	25/27	1.8 (1.7)	3.30 (1.7)	0.88	0.29	30.3	26.1	0	N/A	No	9	Both

deBoer et al. 2021	PC: Number of clauses in utterances	41/40	0.57 (0.02)	0.59 (0.03)	0.73	0.23	28.41	31.7	24.4	423	No	10	Free speech
Schneider et al. 2023	PC: Pure syntactic complexity	34/40	1.43 (0.26)	1.64 (0.28)	0.78	0.24	42.47	40.83	29.4	403	No	10	Free speech
Dalal et al. 2024 (FEP)	PC: Clause complexity	72/39	6.94 (0.68)	7.01 (0.54)	0.11	0.20	22.24	21.79	18	102	Yes	9	Picture description
Dalal et al. 2024 (SCZ)	PC: Clause complexity SCZ	18/39	6.78 (0.56)	7.01 (0.54)	0.40	0.29	28.47	21.79	22.2	435	Yes	9	Picture description
Li et al. 2024	PC: Clauses per utterance	38/25	3.28 (1.44)	6.51 (5.85)	0.76	0.28	37.58	37.07	8	286	No	9	Free speech
Çokal et al. 2018 (FTD)	PI: Errors/ utterance	15/15	0.114 (0.105)	0.07 (0.068)	0.50	0.37	50	45	13.3	N/A	Yes	9	Picture description
Çokal et al. 2018 (nFTD)	PI: Errors/ utterance	15/15	0.11 (0.102)	0.07 (0.068)	0.46	0.37	38	45	33.3	N/A	Yes	9	Picture description
DeLisi 2001 (FEP)	PI: Grammatical mistakes	9/12	1.8 (2)	0.90 (1)	0.57	0.45	23.4	32.6	33.3	N/A	Yes	8	Both
DeLisi 2001 (SCZ)	PI: Grammatical mistakes	29/12	1.3 (2)	0.90 (1)	0.25	0.34	33.8	32.6	24.1	N/A	Yes	8	Both
Fraser et al. 1986	PI: Syntactic/ semantic errors	50/50	30.6 (17.12)	16.92 (8.67)	1.01	0.21	28.1	38.2	30	N/A	Yes	10	Both
Gargano et al. 2022	PI: Syntactic completeness	133/133	38.01 (20.92)	45.90 (18.91)	0.40	0.12	28.93	33.07	39.9	N/A	No	9	Picture description
Morice & Ingram 1982	PI: Syntactic/ semantic errors	34/18	21.68 (11.37)	13.11 (4.51)	0.99	0.31	26.7	31.6	29.4	N/A	Yes	10	Free speech
Perlini et al. 2012	PI: Syntactic completeness	30/30	35.15 (14.08)	46.52 (18.68)	0.69	0.27	39.7	38.53	20	354	No	11	Picture description
Shedlak et al. 1997	PI: Morphological errors	37/17	0.7 (0.6)	1.80 (2.2)	0.68	0.30	33.8	31.9	21.6	N/A	Yes	9	Free speech
Thomas et al. 1996	PI: Syntactic error	38/16	7.52 (5.23)	1.53 (1.32)	1.57	0.33	26.96	26.96	36	N/A	Yes	10	Free speech
Vogel et al. 2009	PI: Syntactic/ semantic errors	15/12	2.33 (1.41)	0.29 (0.76)	1.80	0.46	N/A	N/A	0.2	547	Yes	11	Sentence generation
Morice & Ingram 1982	GC: Percentage sentences with embedding	34/18	45.38 (10.54)	54.42 (10.01)	0.88	0.30	26.7	31.6	29.4	N/A	Yes	10	Free speech

Morice & McNicol 1985	GC: Percentage of sentences with simple structure	17/19	8.86 (5.4)	15.63 (5.9)	1.20	0.36	30	30	29	N/A	Yes	10	Free speech
Thomas et al. 1987	GC: Percentage sentences with embedding	18/10	38.71 (9.1)	44.43 (8.6)	0.65	0.40	24.4	24.1	38.9	N/A	Yes	9	Free speech
Sanders et al. 1995	GC: Percentage of sentences with simple structure	11/11	0.37 (0.04)	0.39 (0.07)	0.35	0.43	N/A	N/A	N/A	N/A	Yes	8	Free speech
Thomas et al. 1996	GC: Percentage of sentences with clausal structure	38/16	54.2 (22.1)	69.90 (9.2)	0.93	0.31	26.96	26.96	36	N/A	Yes	10	Free speech
Kircher et al. 2005	GC: The number of complex sentences spoken	6/6	8.5 (2.8)	13.5 (4.1)	1.42	0.65	34.3	34	0	1042	Yes	10	Free speech
Tavano et al. 2008	GC: Percentage of complex syntax	37/37	52.97 (22.18)	63.07 (13.75)	0.55	0.24	39.73	38.16	70.3	N/A	No	11	Storyboard
Özcan et al. 2017	GC: Percentage of sentences with simple structure	50/50	8.8 (5.4475)	4.26 (2.8675)	1.04	0.21	41.98	41	34	N/A	No	10	Multiple
Schneider et al. 2023	GC: Percentage of sentences with simple structure	34/40	0.35 (0.09)	0.23 (0.08)	1.41	0.26	42.47	40.83	29.4	403	No	10	Free speech
Chaves et al. 2023 (SCZ)	GC: Percentage of sentences with simple structure (Sample 1)	20/20	17.43 (3.8)	15.29 (4.25)	0.53	0.32	34.79	35.05	20	N/A	No	10	Dream and waking reports
Chaves et al. 2023 (FEP)	GC: Percentage of sentences with simple structure (Sample 2)	11/20	18.94 (6.35)	18.875 (6.8)	0.01	0.38	17.395	35.05	20	N/A	No	10	Dream and waking reports
Dalal et al. 2024 (FEP)	GC: Syntactic complexity overall measure	72/39	44.68 (9.29)	44.95 (6.53)	0.03	0.20	22.24	21.79	18	102	Yes	9	Picture description
Dalal et al. 2024 (SCZ)	GC: Syntactic complexity overall measure	18/39	46.71 (11.75)	44.95 (6.53)	0.18	0.29	28.47	21.79	22.2	435	Yes	9	Picture description

NOTES: SD=Standard Deviation. N/A=Not Applicable. CPZ=Chlorpromazine dose (rounded to the nearest milligram). FTD=Formal Thought Disorder. nFTD=No-Formal Thought Disorder. SC=Syntax Comprehension. ED=Error Detection. PL=Production Length. PC=Phrasal Complexity. PI=Production Integrity. GC=Global Complexity. SPM=Sentence Picture Matching. TROG=Test for the Reception of Grammar. Q&A=Question and Answer tests for grammatical comprehension. TCGB =Test di Comprensione Grammaticale per Bambini. MLU=Mean Length of Utterance. MLS=Mean Length of Sentence.
