# Supplementary material for: Relationship between grammar and schizophrenia: a systematic review and meta-analysis
Source: Commun Med (Lond). 2025 Jun 16;5:235. doi: 10.1038/s43856-025-00944-1 (PMC12170843; doi:10.1038/s43856-025-00944-1)
Supplement: Supplementary file 11 — Supplementary Data 8 [file 43856_2025_944_MOESM11_ESM.pdf]

Table S6. Medications, linguistic variables and task details from the included studies.

Author(s), Year	Sex of patients (M/F)	Mean age/ SD of controls	Sex of controls (M/F)	Medication average dose (pts.)	Linguistic variables	Nature of the task
Anand et al. 1994	18/6	24.8 (4.3)	16/8	Most were medicated	Errors in four linguistic categories: semantics, syntax, cohesion, and metaphorical language, along with attention and concentration measures	Identifying errors in a list of sentences
Arslan et al. 2024	29/24	22.98 (3.66)	21/29	222 mg (FEP), 194 mg (FEBD)	Semantic similarity, Generic and POS features, Machine-learning classification	Picture description narrative (TAT pictures)
Bagner et al. 2003	18/9	35.39 (9.72)	17/11	Antipsychotics (typical): 20%<br>Antipsychotics (atypical): 69%<br>Antiparkinsonians: 19%<br>Antidepressants: 33%<br>Mood Stabilizers: 11%<br>Benzodiazepines: 4%	Accuracy and reaction time on different sentence types; Reading span performance	Comprehension of spoken sentences with varying complexity
Barattieri di San Pietro et al. 2022	25/9	48.23 (11.24)	25/9	At least one anti-psychotic medication (M = 2.71, SD = 1.53) for at least 6 months prior to entering the study	Various measures of language and cognitive functions were assessed using the Northwestern Assessment of Verbs and Sentences (NAVS) and Brief Assessment of Cognition in Schizophrenia (BACS).	Sentence generation (with picture primes) and sentence comprehension with picture selection
Barrera et al. 2005	24/7	41 (15)	9/8	All patients on antipsychotic medication (details include: clozapine (n=24), risperidone (n=1), olanzapine (n=2), typical antipsychotic drugs (n=9))	Test of Reception of Grammar (TROG)	Comprehension of spoken sentences (tested using a picture selection task called TROG)
Buck & Penn 2015	N/A	N/A	N/A	N/A	Emotion perception (FEIT, FEDT), Theory of Mind (Hinting Task, TASIT), Attributional style (AIHQ), Social functioning (SSPA)	Narrative of Emotions Task (NET) focusing on emotional and bodily state narratives
Çabuk et al. 2024	21/17	37.97 (7.96)	20/18	504.39 (220.29) mg (ECPZ)	Total completed words, first-person singular pronoun usage, mean sentence length, moving average type-token ratio (MATTR), and parts-of-speech tagging, which examine aspects of spontaneous speech and thought processing.	Speech was elicited using a 20-minute semi-structured interview, designed to prompt spontaneous speech and capture both objective and subjective thought and language disorders.
Chaves et al. 2023	16/4	35.05 (11.21)	9/11	Antipsychotic medication, mood stabilizers, benzodiazepines, antidepressants	Nominal Domain: Null pronouns, especially 3P referential pronouns, showed differences. Sentential Domain: Fewer matrix sentences, and more truncated sentences in SZ narratives.	Descriptive speech generated with dream and non-dream (waking) prompts

Çokal et al. 2018	23/7	45 (13.0)	7/8	Various (clozapine, olanzapine, etc.)	Referential anomalies (including vague and unclear references, third-person anaphor anomalies, and 'general' referential anomalies)<br>Production of noun phrases (NPs) (definite and indefinite)<br>Syntactic complexity (measured by the number of grammatical dependents and embedded clauses)<br>Syntactic errors (e.g., agreement violations, tense violations, missing/wrong dependents, truncated utterances)	Participants narrated a story based on an eight-picture comic strip featuring a cat stealing a fish intended for dinner guests.
Çokal et al. 2019	23/7	45 (13.0)	7/8	N/A	Variables include the accuracy scores for sentence-picture matching (SPM), assessed overall and separately for factive and nonfactive conditions.	Sentence Picture Matching and TROG
Condray et al. 1995	15/0	35.2	15/0	Haloperidol and placebo on assessments	Language comprehension: evaluating the understanding of logical relationships expressed through syntactic connectives, word order, and interclausal relations.	Relational Concepts Factor Scale (R2 from the Luria-Nebraska Scale Form 1) Errors
Condray et al. 2002	32/0	38.9 (8.4)	22 M	504.02 mg/day in chlorpromazine equivalents	Comprehension accuracy for sentence processing (simple declarative, relative sentences), Sentence Span Test, Intelligibility task.	Comprehension of spoken sentences with varying complexity
Dalal et al. 2024	72/18	21.79 (3.47)	26/13	DDD for antipsychotics: FEP (0.34), SZ (1.45)	Syntactic and morphological features include analytic thinking index, linguistic function words, syntactic complexity, and clause complexity.	Picture description narrative (3 TAT pictures)
de Boer et al. 2021	31/10	31.7 (11.71)	36/4	High D2R: 347.8 mg, Low D2R: 518.6 mg	Praat software-based acoustic and morphological variables	Speech from semi-structured interview responses
DeLisi 2001	28/10	32.6 (7)	7/5	Conventional antipsychotics, Haloperidol vs. Risperidone (research protocol for some patients)	Language and speech abnormalities (syntax, semantics, fluency, etc.)	
Delvecchio et al. 2019	76/90	31.8 (9.0)	48/58	245.5	Syntactic comprehension using the TCGB test	Sentence interpretation with picture matching
Dwyer 2014	24/8	35.9 (14.8)	9/6	N/A	Sense judgment of sentences - single and pairs	Comprehension of spoken sentences
Fraser et al. 1986	35/15	38.2 (14.17)	24/26	N/A	Variables include mean depth of embedding, dysfluency index, total error count, and number of embedded clauses per complex sentence.	Free speech (analysis of first 1000 words)
Gargano et al. 2022	80/53	33.07 (9.56)	64/69	N/A	Several language production variables at micro- and macro-linguistic levels.	Picture description narrative
King et al. 1990	N/A	33.1 (15.12)	N/A	High (not specified)	Various linguistic measures based on PSYCH-LAN	Language analysis of free speech
Kircher et al. 2005	6/0	N/A	6M	1042 mg/day, SD=738	Simple sentences: sentences consisting of a subject and predicate, containing one finite verb. Complex sentences:	Picture description narrative

					sentences consisting of one independent clause together with one or more subordinate clauses. Number of simple and complex sentences evaluated per 20-second epoch.	
Kuperberg et al. 2006 (1)	17/3	41 (8)	18/2	410	Reading and acceptability of decision time differences across normal, semantic/pragmatic, animacy, and morphosyntactically violated sentences	Acceptability judgments on written sentences
Kuperberg et al. 2006 (2)	15/5	41 (12)	16/4	467	Event-related potentials to critical verbs were measured as patients with SZ and healthy controls read sentences word by word.	Acceptability judgments on written sentences
Lee et al. 2016	16/10	33.6 (5.3)	12/17	654.17	Normal and ungrammatical auditory sentences to judge syntactic intactness	Comprehension of spoken sentences
Li et al. 2024	35/3	37.07 (9.41)	18/7	286.36 mg/day (Chlorpromazine equivalent)	SPR is higher in HCs, DF is higher in SZ patients. Combined SPR + DF achieved 84.5% accuracy in classifying FTD.	Free-speech task, PANSS interviews
Liang et al. 2022	54/12	21.15 (3.08)	24/12	Less than 14 days of lifetime exposure to antipsychotic medications for patients	TLI: reduced total score, impoverishment, disorganization; MLS, MLT, and MLC significantly reduced in Subgroup 2 compared to controls and Subgroup 1	Picture description narrative
Morgan et al. 2021	N/A	N/A	N/A	N/A	TLI negative score related to the number of words, LCC, LCCr, and LSC, LSCr; NLP measures did not correlate with TLI	Describing pictures from the TAT, retelling stories from the DCT, and engaging in free speech on a self-selected topic for 10 minutes.
Morice & Ingram 1982	24/10	31.6 (12.3)	10/8	Widely-ranging doses of antipsychotic medication	Embedded clauses, sentence complexity, grammatical errors, dysfluency (PSYCH-LAN)	Free speech 1000 words
Morice and McNicol 1985	N/A	mentioned as closely matched to patients	N/A	Equivalent amounts of antipsychotic medication are measured in Chlorpromazine equivalents.	Reduced comprehension and production of complexity in Sc and BP (PSYCH-LAN)	Comprehension of spoken sentences and Free speech (1000 words)
Moro et al. 2015	32/26	37.93 (11.95)	11/19	Clozapine (median daily dose 250 mg, 41 patients)<br>Haloperidol (median daily dose 3.5 mg, 11 patients)<br>Risperidone (median daily dose 3 mg, 3 patients)<br>Olanzapine (median daily dose 7.5 mg, 3 patients)	Detecting syntactic and semantic set anomalies across short and long sentences.	150 short/long sentences followed by questions on comprehension
Özcan et al. 2017	33/17	41 (3.30)	33/17	N/A	Higher frequency of syntactically simple sentences	Picture description, semistructured interview, free speech, and picture sequencing tasks
Panikratova et al. 2021	25/0	26.1 (4.8)	27M	N/A	Text length, mean phrase length, number of clauses, programming, grammatical formulation, semantic completeness, independence	Story generation with and without pictures

Perlini et al. 2012	24/6	38.53 (12.71)	17/13	11 typical, 18 atypical antipsychotics; some patients on additional antipsychotic medication	Reduced MLU, speech rate, increased paragrammatic errors, impaired syntactic completeness	Story generation and written sentence interpretation with multiple picture-based choices
Sanders et al. 1995	N/A	N/A	N/A	N/A	Productivity, repetition, dysfluency, cohesiveness, and syntactic complexity (MLU, number of complex, simple sentences, coordinated clauses, etc)	Picture description narrative
Schneider et al. 2023	24/10	40.83 (13.35)	15/25	402.54 (773.65).	Syntactic complexity and diversity along with verbal fluency.	Picture description narrative
Sevilla et al. 2018	24/16	39.6 (10.83)	8/6	SZ+TD: Chlorpromazine equivalents: 1072.05 mg (595.45) SZ-TD: Chlorpromazine equivalents: 624.57 mg (317.32)	Anomalies in definite NPs, pronouns, 3rd person NPs, anomalies in indefinite NPs, NPs involving lexical nouns, complement clauses, semantic selectional restrictions, formal grammatical errors, and grammatical dependents per utterance.	Story generation (fairytale)
Shedlack et al. 1997	29/5	31.9 (4.7)	9/8	26 patients were on antipsychotic medications, and 13 were also using anticholinergics.	Linguistic complexity, verbal fluency	6 min free speech
Stephane et al. 2007	20/2	47 (12)	8/3	308	Impairment in recognizing incorrect stimuli at the syntactic and other higher levels of language processing.	Identifying errors in written sentences
Stirling et al. 2006	18/12	36.22 (9.32)	N/A	Neuroleptic medication, average daily dose 573 mg CPZE	Syntactic comprehension and semantic fluency	Comprehension of spoken sentences (tested using a picture selection task called TROG)
Tan et al. 2016	30/27	39.83 (13.89)	20/28	CPZE= 477.82 (429.77)	Error responses in lexical recognition, synonym identification, and sentence comprehension tasks.	Comprehension of meaning from 56 pairs of spoken sentences
Tang et al. 2021	11/9	35.6 (5.8)	4/7	N/A	NLP-based measures of POS, sentence coherence	Open-ended interviews
Tavano et al. 2008	11/26	38.16 (11.80)	16/21	Patients were on antipsychotic medications at the time of assessment.	Narratives, syntactic comprehension, pragmatic comprehension	Story generation and free speech (100 utterances)
Thomas et al. 1987	11/7	24.1 (5.8)	6/4	N/A	Complexity, integrity, and fluency of speech	Free speech (1000 words)
Thomas et al. 1996	N/A	18.4 (5.3)	N/A	Mean for SZ = 19.5, Mean for Mania = 13.0	Mean length of utterance, percentage of well-formed major sentences, mean maximum depth of embedding, percentage of deviant sentences, and syntactically deviant sentences.	Free speech (1000 words)
Vogel et al. 2009	12/3	N/A	9/3	Antipsychotic treated	Semantic knowledge, lexical-semantic access, and retrieval accuracy.	Sentence Generation from single-word prompts.

NOTES: SZ=Schizophrenia. HC=Healthy Controls. FEP=First Episode Psychosis. FEBD=Bipolar Disorder. SSEM=Total number of Semantic errors. SSYN=Total number of syntactic errors. SCOH=Total number of cohesion errors. SMET=Total number of metaphorical language errors. AVC=AVerage Concentration deficit (measured with the Forward Digit Span Task, FDST). BPRS=Brief Psychiatric Rating Scale. TROG=Test for the Reception of Grammar. DDD=Mean Daily Defined Dose. MLU=Mean Length of Utterance. MLS=Mean Length of Sentence. MLT=Mean Length of Turn. MLC=Mean Length of Clause. CPZE=Chlorpromazine equivalents. NP=TLC=Thought, Language, and Communication. TAT=Thematic Apperception Test. SPM=Sentence Picture Matching. DCT=Discourse Comprehension Test. TCGB=Test di Comprensione Grammaticale per Bambini. POS=Parts of Speech. NLP=Natural Language Processing. NP=Noun Phrase. SANS=Scale for the Assessment of Negative Symptoms. PANSS=Positive and Negative Syndrome Scale. BPRS=Brief Psychiatric Rating Scale. FTD=Formal Thought Disorder. nFTD=no-Formal Thought Disorder. SLOF=Specific Levels of Functioning Scale. ToM=Theory of Mind. TALD=Thought and Language Disorder Scale. TDI=Thought Disorder Index. SAPS=Structured Interview Guide for the Assessment of Negative Symptoms; BNSS=Brief Negative Symptom Scale; YMRS=Young Mania Rating Scale; HDRS=Hamilton Depression Rating Scale. PSE=Present State Examination. SAPS=Scale for the Assessment of Positive Symptoms. RDC=Research Diagnostic Criteria. TALD=Thought and Language Disorder.
